# RGD-independent binding of Russell’s Viper venom Kunitz-type protease inhibitors to platelet GPIIb/IIIa receptor

**DOI:** 10.1038/s41598-019-44767-2

**Published:** 2019-06-05

**Authors:** Bhargab Kalita, Sumita Dutta, Ashis K. Mukherjee

**Affiliations:** Microbial Biotechnology and Protein Research Laboratory, Department of Molecular Biology and Biotechnology, Tezpur University, Tezpur, 784028 Assam India

**Keywords:** Haematological diseases, Proteins

## Abstract

This study elucidates the platelet-modulating properties of two snake venom Kunitz-type serine protease inhibitors, Rusvikunin and Rusvikunin-II, from Russell’s Viper venom, their native and reconstituted complexes, and two synthetic custom peptides (developed from the platelet-binding region of Rusvikunin-II) against mammalian platelet-rich plasma (PRP) and washed platelets. The Rusvikunins and their complexes demonstrated concentration-dependent deaggregation and aggregation of washed platelets independent of von Willebrand factor and/or fibrinogen requirement. At lower concentrations they abolished collagen and ADP-induced platelet aggregation, but at higher concentrations, they progressively decreased the inhibition of ADP-induced aggregation and potentiated the effect of collagen on PRP. Rusvikunin complex/Rusvikunin-II bound to and induced RGD-independent aggregation of α-chymotrypsin-treated platelets. Molecular docking studies suggested interaction of Rusvikunin-II and custom peptides with platelet GPIIb/IIIa receptor, which was validated by spectrofluorometry analysis and ELISA. This study reports, for the first time, an RGD-independent binding of a snake venom component to the platelet GPIIb/IIIa receptor.

## Introduction

Snake envenoming is an occupational health hazard that affects as many as 2.7 million individuals every year primarily in the developing countries of Asia, Africa, Latin America, and Oceania^1,2^. In 2017, the World Health Organization listed snake envenoming as a priority neglected tropical disease and in May 2018, the 71^st^ World Health Assembly endorsed a strong resolution on snake envenoming with an aim to give greater priority to this disease of poverty and its victims^2^. Russell’s Viper (RV; *Daboia russelii*) accounts for a large amount of mortality and morbidity in South and Southeast Asian countries, and is considered as a category I medically important snake in India^3^. RV venom (RVV) is primarily hemotoxic, affecting the blood vascular system by provoking haemostatic disturbances, including rapid thrombosis and hypofibrinogenemia that ultimately results in consumption coagulopathy and incoagulable blood^3–5^. These clinical manifestations are primarily from the concerted action of pro-coagulant proteases, anticoagulant phospholipase A_2_ enzymes, and Kunitz-type serine protease inhibitors (KSPI)^3,6^. Proteomic studies on RVV from different geographic locations in the Indian sub-continent have shed light on the geographical variation of RVV composition and the associated clinical manifestations of RV envenomation in different locales^7–11^.

Snake venom components often interact to form stable/cognate complexes^12–14^ and bind specifically to target molecule(s) thereby augmenting their toxicity, biological activity, and pharmacological propensity^13,14^. Our previous studies have shown that Rusvikunin^15^ and Rusvikunin-II^16^, the two Kunitz-type serine protease inhibitors (KSPI) purified from the venom of *D*. *russelii* of Pakistan origin, non-covalently interact at a 1:2 stoichiometric ratio to form a snake venom protein complex called Rusvikunin complex^17^. The formation of the complex with KSPIs preferentially augments the pharmacological properties beyond those of the individual components of the complex^16^.

Hemostasis is a complex and highly organized process that responds to disruption of the vascular endothelium^18^. Further, coagulation factors and blood platelets is orchestrated during the hemostatic response to prevent loss of blood from an external injury^19^. Many of the coagulation factors bind to activated platelets via glycoprotein receptors or plasma phospholipids, leading to several responses that counter blood loss^19^. Interestingly, RVV components have been demonstrated to interfere with these interactions, eventually conferring hemostatic disturbances in bite victims or prey^20,21^.

The platelet membrane glycoprotein IIb/IIIa (GPIIb/IIIa) receptors play a vital role during hemostasis by regulating platelet adhesion and aggregation^18^. Fibrinogen and fibronectin, via binding to GPIIb/IIIa receptors link the aggregating platelets together to stabilize the platelet plug^19^. The binding of fibrinogen to the integrin receptor has been shown to be Arg-Gly-Asp (RGD)-dependent^22^; however, fibronectin exhibits both RGD-dependent and independent binding to the receptors^23^. In-depth studies have further mapped the residues Ile^1359^ to Ser^1436^ and Ala^1597^ to Glu^1963^ of fibronectin as being involved in binding to the GPIIb/IIIa receptor in an RGD sequence-independent manner^23,24^. In addition, synthetic peptides corresponding to these regions have also demonstrated binding to immobilized GPIIb/IIIa receptors^24^.

The present study investigates the platelet modulation properties of Kunitz-type protease inhibitors and their putative protein complexes isolated from snake venom for the first time. This report is also the first to show RGD sequence-independent binding of RVV components and their complexes with the platelet GPIIb/IIIa receptor to modulate platelet function.

## Results and Discussion

### Platelet modulating activity of native and reconstituted Rusvikunin complexes and their components

Platelet functions are altered by snake venom proteins via binding, blocking, clustering, activating, or by cleaving platelet receptors or the von Willebrand factor^21,25–29^. Although the platelet-modulating activity of several components of snake venom has been well explored, to date, this property has not been documented for the snake venom Kunitz-type protease inhibitors. To the best of our knowledge, this report is the first to show platelet-modulating activity of snake venom Kunitz-type protease inhibitors isolated from RVV.

**Figure 1:**
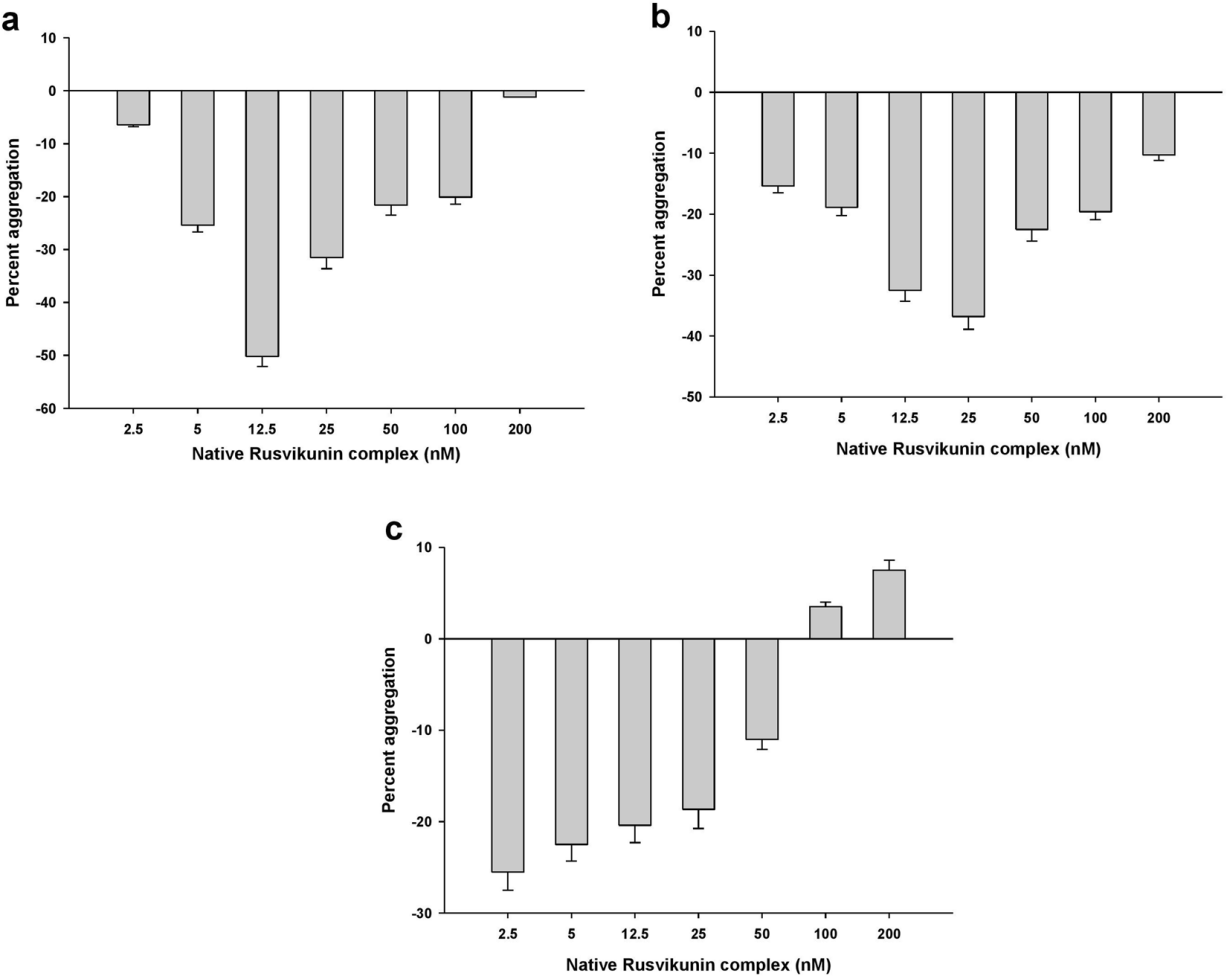
Concentration-dependent platelet modulating activity of native Rusvikunin complex on PRP from (**a**). Goat blood and (**b**). Human blood. Values are mean ± SD of triplicate determinations. (**c**) Concentration-dependent platelet modulating activity of native Rusvikunin complex on washed platelets from goat blood. Values are mean ± SD of triplicate determinations.

Rusvikunin, Rusvikunin-II, and reconstituted or native Rusvikunin complexes (isolated from crude RVV) demonstrated concentration-dependent deaggregation of PRP from goat (Fig. 1a, Supplementary Fig. S1a,b) and human blood (Fig. 1b, Supplementary Fig. S1c,d). Notably, the extent of deaggregation of PRP was progressively increased with an increasing concentration of the proteins/complex to 12.5 nM (Rusvikunin complex) or 37.5 nM (Rusvikunin or Rusvikunin-II) for goat PRP and to 25 nM (Rusvikunin complex) or 75 nM (Rusvikunin or Rusvikunin-II) for human PRP. However, with a further increase in concentration (>12.5 nM for goat PRP and >25 nM for human PRP), the extent of platelet deaggregation decreased concomitantly (Fig. 1a,b; Supplementary Fig. S1a–d). Further, the differential deaggregation of goat and human platelets by the same concentration of Rusvikunin complex or native Rusvikunins may be correlated to their higher binding to goat platelets (Supplementary Fig. S2). ATP also activates or inhibits platelet function depending on the degree of purinergic P2Y_1_ and P2X_1_ receptor occupancy^30^. Therefore, the Rusvikunins or Rusvikunin complex would also be expected to bind to two different receptors in a concentration-dependent manner, to function as an antagonist or agonist^21,30,31^. Our findings indicate that the Rusvikunin complex-induced platelet modulation results from an equilibrium of aggregation and deaggregation processes that may depend on sub-receptor occupancy by the Rusvikunin complex or its components (see below).

The reconstituted and native Rusvikunin complexes also demonstrated concentration-dependent deaggregation and aggregation of mammalian washed platelets (Fig. 1c; Supplementary Fig. S3). This data suggests that, similar to aggretin isolated from the venom of *Calloselasma rhodostoma* (Malayan Pit Viper) that induces potent platelet aggregation, the platelet modulating activity of the Rusvikunin complex is independent of von Willebrand factor (vWF) and/or fibrinogen requirement^32^. In this regard, the Rusvikunins or Rusvikunin complex differs from other platelet-modulating components of snake venom that require fibrinogen and/or vWF to induce aggregation of washed platelets^33,34^. Fibrinogen and vWF are platelet agonists that selectively bind to platelet GPIIb/IIIa and GPIb receptors, respectively. Upon limited proteolytic activation, however, GPIIb/IIIa can also bind to fibronectin, resulting in irreversible platelet adhesion^35^.

### Effect on chymotrypsin-treated platelet

The GPIIb/IIIa receptor remains in an inactive conformation on resting platelets and serves as a low-affinity adhesion receptor for surface-coated fibrinogen. Nevertheless, upon platelet activation by strong (collagen, thrombin) or weak (epinephrine, adenosine diphosphate) agonists, GPIIb/IIIa undergoes a conformational change in its quaternary structure, which leads to the reorientation of its ligand-binding cavity and subsequent high-affinity binding of macromolecular ligands such as fibrinogen^36^. Our observation that fibrinogen induces aggregation of α-chymotrypsin-treated platelets, albeit it has no effect on control (untreated) platelets (Table 1) is correlated with the fact that soluble fibrinogen does not bind to untreated washed platelets. Nevertheless, the limited proteolysis with α-chymotrypsin exposes the two subunits of the platelet fibrinogen receptor, GPIIb and GPIIIa, on the surface of platelets without interfering with cell activation or granular secretion^37,38^. Consequently, fibrinogen can bind to platelets treated with α-chymotrypsin and cause aggregation of platelets^37,38^. Interestingly, at lower concentrations the reconstituted and native Rusvikunin complexes (2.5 nM) and Rusvikunin-II (7.5 nM) also provoked aggregation of α-chymotrypsin-treated platelets (Table 1), but not the untreated (control) platelets (Fig. 1a–c; Supplementary Fig. S1a–d). Pre-incubation of α-chymotrypsin-treated platelets with monoclonal antibody against the GPIIb/IIIa receptor resulted in a complete abolition of the fibrinogen- and Rusvikunin complex/Rusvikunin-II-mediated platelet aggregation (Table 1). These results provide convincing evidence that similar to fibrinogen, the reconstituted/native Rusvikunin complex and Rusvikunin-II (the major constituent of the Rusvikunin complex) also binds to the exposed fibrinogen-receptor GPIIb/IIIa on the platelet surface. Moreover, the degree of platelet aggregation is dependent on the high-affinity fibrinogen receptor occupancy^37^. Furthermore, BSA (negative control) did not induce aggregation of the α-chymotrypsin-treated platelets, presumably because of the lack of affinity for the exposed GPIIb/IIIa receptor (Table 1). Nevertheless, the monoclonal antibody against the GPIIb/IIIa receptor could not inhibit the concentration-dependent platelet aggregation (200 and 400 nM) or the deaggregation (2.5 and 5.0 nM) shown by native Rusvikunin complex in α-chymotrypsin-untreated platelets (Supplementary Fig. S4). These findings suggest that, like fibrinogen, the interaction of higher concentrations of Rusvikunin complex with the GPIIb/IIIa receptor on resting platelets (see below) may not induce platelet aggregation.

**Table 1:** Percent aggregation of α-chymotrypsin-treated or untreated washed platelets (1 × 10^6^ cells/mL) by different agonists. Values are mean ± SD of four determinations.

Agonist	Treatment with α-chymotrypsin (8 U/mL, 15 min, RT)	Treatment with mAb_GPIIb/IIIa_ (10 µg/mL, 30 min, RT)	% platelet aggregation
Fibrinogen (175 nM)	No	No	Nil
Fibrinogen (175 nM)	Yes	No	15.9 ± 2.0
Fibrinogen (175 nM)	Yes	Yes	0.9 ± 0.01
Reconstituted Rusvikunin complex (2.5 nM)	Yes	No	8.1 ± 1.1
Reconstituted Rusvikunin complex (2.5 nM)	Yes	Yes	(−) 13.2 ± 0.7
Native Rusvikunin complex (2.5 nM)	Yes	No	9.4 ± 1.2
Native Rusvikunin complex (2.5 nM)	Yes	Yes	(−) 14.8 ± 0.8
Rusvikunin II (7.5 nM)	Yes	No	5.2 ± 0.8
Rusvikunin II (7.5 nM)	Yes	Yes	(−) 1.2 ± 0.03
RusPep (5 nM)	Yes	No	(−) 4.1 ± 0.05
RusPep (5 nM)	Yes	Yes	(−) 3.7 ± 0.07
CycRusPep (5 nM)	Yes	No	3.1 ± 0.09
CycRusPep (5 nM)	Yes	Yes	(−) 2.8 ± 0.08
BSA (1 µM)	Yes	No	Nil

mAb_GPIIb/IIIa_ - monoclonal antibody against platelet GPIIb/IIIa receptor, RT - room temperature (~23 °C), (−) percentage of platelet aggregation signifies platelet deaggregation property of the agonist.

**Figure 2:**
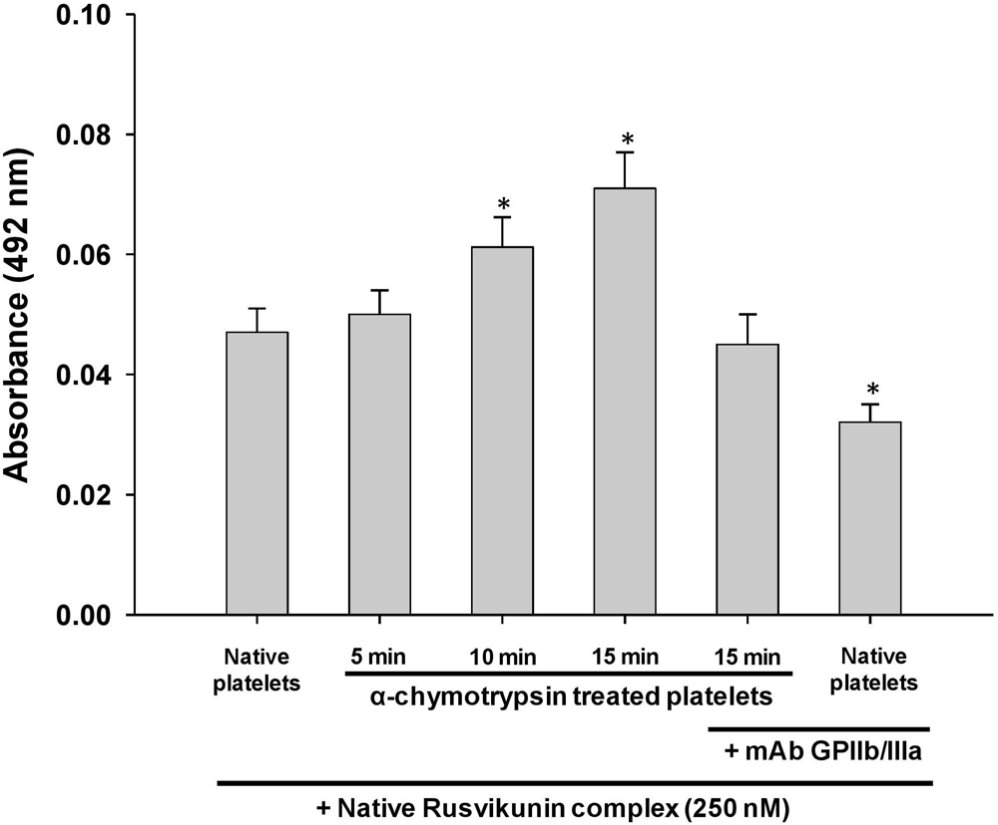
Determination of binding of native Rusvikunin complex (250 nM) on α-chymotrypsin-treated or untreated washed platelets (1 × 10^6^ cells/mL) by ELISA. Experimental details are described in the text. Values are mean ± SD of six determinations. Significance of difference with respect to Native platelets, *p < 0.05.

The binding of native and reconstituted Rusvikunin complex and Rusvikunin-II to washed platelets progressively increased with increased treatment time of α-chymotrypsin (Fig. 2; Supplementary Fig. S5). Pre-incubation of platelets with monoclonal antibody against the GPIIb/IIIa receptor complex completely inhibited this enhanced association of Rusvikunin-II or Rusvikunin complex to the platelet surface (Fig. 2; Supplementary Fig. S5). This result indicates that exposure to the receptors of fibrinogen on platelet membrane leads to a greater binding of the Rusvikunin complex/Rusvikunin-II most likely through interactions of this complex with the GPIIb/IIIa receptor. Notably, the binding of Rusvikunin complex/Rusvikunin-II to native platelets was partially inhibited by the monoclonal antibody against the GPIIb/IIIa receptor (Fig. 2; Supplementary Fig. S5), which suggests that this protein/complex can bind to inactivated GPIIb/IIIa receptors on the platelet surface, though, as already discussed, this binding may not lead to platelet aggregation. Previously, the RGD sequence (Arg^1493^ to Ser^1496^) that is present in fibrinogen, fibronectin, and many other snake venom proteins have been demonstrated to bind to the GPIIb/IIIa integrin receptor^39^. Nevertheless, RVV Kunitz-type protease inhibitors lack the RGD sequence in their primary structure^15,40^ suggesting that binding of the Rusvikunin complex to GPIIb/IIIa does not involve the RGD sequence. Although the RGD-independent binding of proteins and peptides other than from snake venom origin to the GPIIb/IIIa receptor has been demonstrated^23,32,39,41^, our report is first to show the RGD-independent binding of a snake venom Kunitz-type serine protease inhibitor to the platelet GPIIb/IIIa receptor.

### Effect on collagen and ADP-induced platelet aggregation

**Figure 3:**
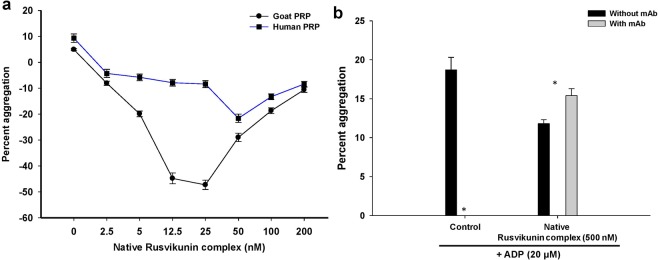
(**a**) Effect of different concentrations of native Rusvikunin complex on ADP (20 μM)-induced aggregation of goat and human PRP. Values are mean ± SD of six determinations. (**b**) Effect of ADP (20 μM) or native Rusvikunin complex (500 nM) on human washed platelets (1 × 10^6^ cells/mL) pre-incubated with monoclonal antibody (10 μg/mL) against ADP P2Y_12_ receptor or buffer (1X PBS, pH 7.4) for 30 min at ~23 °C. The monoclonal antibody did not induce platelet aggregation. Values are mean ± SD of six determinations. Significance of difference with respect to platelet aggregation in presence of mAb against P2Y_12_ receptor, *p < 0.05.

A number of mechanisms could explain how a particular component of snake venom can modulate platelets. For example, proteases may activate platelets by digestion of collagen^42^, by degradation, or through interacting with different platelet receptors^43,44^. The non-enzymatic venom proteins such as disintegrins or snaclecs modulate platelet function by binding to integrin or glycoprotein receptors on their surface^25,26^. Although reconstituted/native Rusvikunin complex and Rusvikunin-II can dose-dependently abolish the ADP-induced aggregation of goat PRP, this deaggregation effect is dependent on the concentration of antagonist (Fig. 3a; Supplementary Fig. S6a,b). The inhibition of ADP-induced aggregation increased up to a 25 nM concentration of reconstituted/native Rusvikunin complex or 75 nM of Rusvikunin-II, though a further increase in concentration decreased the inhibition of ADP-induced aggregation of goat and human PRP (Fig. 3a; Supplementary Fig. S6a,b). The reconstituted/native Rusvikunin complex or Rusvikunin-II followed a same trend with human PRP, but to a different extent (Fig. 3a; Supplementary Fig. S6a,b). ADP also induced aggregation of washed human platelets (Fig. 3b). Pre-incubation of washed platelets with monoclonal antibody against P2Y_12_ receptor completely abolished the ADP-induced aggregation of platelets, while significantly enhancing the platelet aggregation property of the reconstituted/native Rusvikunin complex (Fig. 3b; Supplementary Fig. S6c). Further, the inhibition of ADP-induced aggregation by low concentration of Rusvikunin complex (2.5 and 5.0 nM) was abolished by the monoclonal antibody against P2Y_12_ (Supplementary Table S1).

The above results indicate that at lower concentrations, both the Rusvikunin complex and Rusvikunin-II interferes with ADP binding to its P2Y_1_ and/or P2Y_12_ receptors that lead to the suppression of platelet aggregation^31^. Nevertheless, at higher concentrations (or after blocking the P2Y_12_ receptor of ADP with monoclonal antibody), Rusvikunin-II or the Rusvikunin complex progressively bind to some unidentified receptor(s) on the platelet surface that results in a gradual aggregation of platelets^31^. Further studies are warranted to identify the receptors.

**Figure 4:**
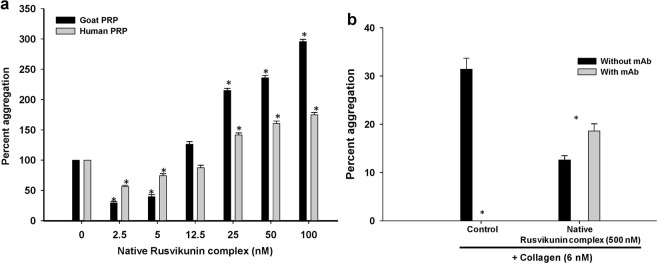
(**a**) Dose-dependent effect of native Rusvikunin complex on collagen (6 nM)-induced aggregation of goat and human PRP. The aggregation induced by collagen was considered as 100% aggregation and the other values were compared to that. Significance of difference with respect to platelet aggregation induced by collagen only; *p < 0.05. (**b**) Effect of collagen (6 nM) or native Rusvikunin complex (500 nM) on human washed platelets (1 × 10^6^ cells/mL) pre-incubated with monoclonal antibody (10 μg/mL) against collagen GPVI receptor or buffer (1X PBS, pH 7.4) for 30 min at ~23 °C. The monoclonal antibody did not induce platelet aggregation. Values are mean ± SD of six determinations. Significance of difference with respect to platelet aggregation in presence of mAb against GPVI receptor, *p < 0.05.

Interestingly, the reconstituted/native Rusvikunin complex also showed different responses in the collagen-induced aggregation of human and goat PRP. At lower concentration ranges (2.5–25 nM and 2.5–5 nM for human and goat PRP, respectively), the complexes partially reserved the collagen (type IV)-induced aggregation of human/goat PRP with decreasing orders of magnitude (Fig. 4a; Supplementary Fig. S7a). At higher concentrations (>25 nM); however, the reconstituted/native Rusvikunin complex dose-dependently potentiated aggregation of goat or human PRP induced by collagen in a dose-dependent manner (Fig. 4a; Supplementary Fig. S7a). Although collagen has been shown to bind to various receptors, such as GPVI, α2β1, p65, p47, TIIICBP, and GPIV on the platelet surface, platelet activation is highly influenced by its binding to integrin α2β1 and GPVI receptors^45,46^. Pre-incubation of washed platelets with monoclonal antibody against the collagen GPVI receptor completely abolished the collagen-induced aggregation of platelets, but significantly (p < 0.05) enhanced platelet aggregation induced by a higher concentration (500 nM) of the Rusvikunin complexes (Fig. 4b; Supplementary Fig. S7b). On the contrary, pre-incubation of platelets with the monoclonal antibody against GPVI receptor abolished the inhibition of collagen-induced aggregation of platelets by a lower concentration of Rusvikunin complex (2.5 and 5.0 nM) (Supplementary Table S1). Since the Rusvikunin complexes cannot degrade collagen (data not shown), they are inferred, at lower concentrations, to act as partial antagonist to the collagen GPVI receptors or bind to a specific collagen receptor leading to partial suppression of collagen-induced aggregation of platelets. Nevertheless, incremental occupancy of some other receptor subtypes on the platelet membrane by higher concentrations (>25 nM) of the Rusvikunin complex or by blocking the GPVI receptor of platelets may attenuate the above inhibition and gradually potentiate platelet aggregation induced by collagen. Elucidating the exact mechanism of such platelet modulation by the Rusvikunin complex is our next goal of study.

### Identification of the GPIIb/IIIa receptor binding sequence lacking the RGD domain in Rusvikunins

Fibronectin has been reported to bind to the GPIIb/IIIa receptor in both RGD-dependent and independent manners^23^. The sequence stretch Ile^1359^ to Ser^1436^ and a 15-residue peptide representing residues from Ala^1704^ to Glu^1718^ of fibronectin that lack an RGD sequence have been demonstrated to bind to the GPIIb/IIIa receptor^23,24,41,47^. Further, a radio-labeled 12-residue synthetic peptide ^1371^REDRVPHSRNSI^1382^ representing a part of the Ile^1359^ to Ser^1436^ region of fibronectin has been shown to inhibit the association of fibrinogen and fibronectin to the integrin receptor GPIIb/IIIa^24^. When this stretch of the fibronectin sequence (Ile^1359^ to Ser^1436^) is aligned with Rusvikunin-II (the major component of the Rusvikunin complex) using the EMBOSS Needle (Global alignment; Needleman-Wunsch algorithm), a 12.6% identity is observed between the sequences with a score of 2.5 (Supplementary Fig. S8a). Nevertheless, when the alignment is switched to local alignment, using Emboss Water (Smith-Waterman algorithm), the sequence ^1363^PEHFSCRPREDRVPHSRNS^1381^ of fibronectin shares 31.6% identity with ^10^PESGRCRGHLRRIYYNLES^28^ of Rusvikunin-II, with a score of 24.0 (Supplementary Fig. S8b). Therefore, Rusvikunin-II may exhibit RGD-independent binding with the GPIIb/IIIa receptor via the above sequence stretch, which was confirmed by the spectrofluorometry analysis (see below).

**Figure 5:**
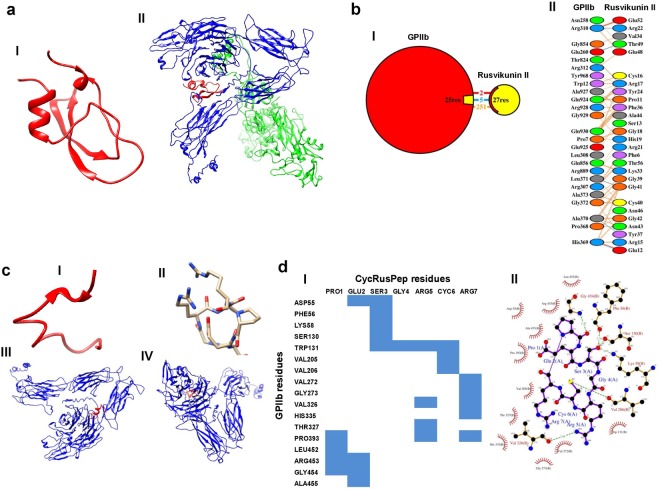
(**a**) Best predicted structure for Rusvikunin-II by the I-TASSER server (panel I) and best docking model of Rusvikunin-II (red color) and human GPIIb/IIIa (blue-colored GPIIb subunit and green-colored GPIIIa subunit) as predicted by the PatchDock server and refined by the Firedock server (panel II). (**b**) Schematic representation of the interaction of Rusvikunin-II with the GPIIb subunit showing the different types of bonds formed between Rusvikunin-II and GPIIb (panel I), and residue-to-residue interactions (panel II) between the two chains, as predicted by the PDBSum server. Salt bridges (ionic), H-bonds, and non-bonded contacts are represented by red, blue, and orange-colored lines, respectively. (**c**) Best predicted structures for RusPep and cycRusPep by the PEP-FOLD3 and PEPstrMOD servers, respectively (panels I and III) and best docking models of RusPep (red color) and cycRusPep (red color) with the GPIIb subunit (blue color) of the platelet GPIIb/IIIa receptor as predicted by the PatchDock server and refined by the Firedock server (panels II and IV). (**d**) Contact-map analysis between the residues of GPIIb (vertical axis) and cycRusPep (horizontal axis), as predicted by the PDBSum server (panel I) and Ligplot analysis to show the residue-to-residue interactions of cycRusPep with GPIIb as predicted by the PDBSum server (panel II).

Docking studies suggest that the best predicted structure of Rusvikunin-II (Fig. 5a, panel I) interacts with the GPIIb subunit of the platelet GPIIb/IIIa receptor via 2 salt bridges, 5 hydrogen bonds, and 251 non-bonded contacts (Fig. 5b). Further, 27 amino acid residues of Rusvikunin-II interacted with 25 amino acid residues of the GPIIb subunit of the fibrinogen receptor (Fig. 5b). Interestingly, among these 27 residues of Rusvikunin-II, 11 residues lie within the region of Rusvikunin-II from which the custom peptide, RusPep was synthesized. Nevertheless, when the best predicted structure of RusPep (Fig. 5c, panel I) was docked with the GPIIb/IIIa receptor, only 112 non-bonded contacts were found between the two molecules (Fig. 5c, panel II). This suggests that the interaction between RusPep and GPIIb/IIIa may be weaker than that of its parent molecule Rusvikunin-II possibly because of their different 3-D structures. On the contrary, the best predicted structure of the cyclic peptide (cycRusPep) (Fig. 5c, panel III) exhibited profound interactions with the GPIIb subunit of the platelet receptor via 6 hydrogen bonds and 113 non-bonded contacts (Fig. 5c, panel IV), which suggests that the appropriate 3-D structure of the custom peptides is important for establishing an interaction with the receptor molecule. Further, the contact map and Ligplot analyses suggested that 17 residues of the GPIIb subunit interacted with all 7 residues of cycRusPep (Fig. 5d). Taken together, the findings from the *in silico* studies suggest that the binding and interaction of Rusvikunin-II and its derived custom peptides with the platelet GPIIb/IIIa receptor induce platelet aggregation.

### Spectrofluorometry study shows interactions between the Rusvikunin complex/custom peptides and the GPIIb/IIIa receptor protein

**Figure 6:**
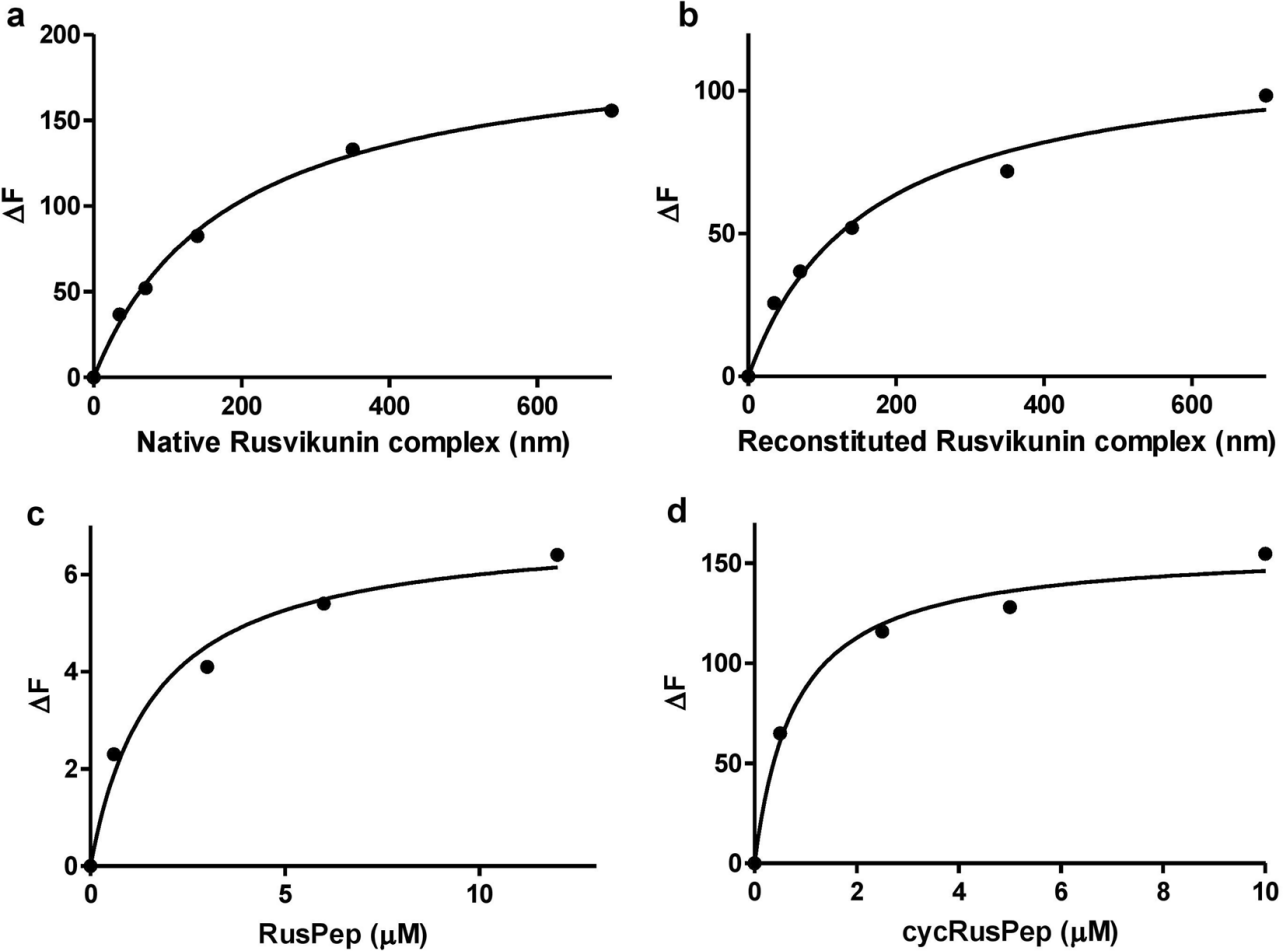
One site-specific binding curves of spectrofluorometry analyses showing the change in maximum fluorescence intensity (λmax) of human GPIIb/IIIa (6 nM) in the presence of different concentrations of (**a**). Native Rusvikunin complex (35–700 nM), (**b**). Reconstituted Rusvikunin complex (35–700 nM), (**c**). RusPep (0.6–12 µM), and (**d**). cycRusPep (0.5–10 µM).

Fluorescence spectrometry is an important technique for determining the interactions between proteins by measuring the intrinsic fluorescence imparted by their aromatic residues^17,20,48,49^. The spectrofluorometry study demonstrated that the reconstituted/native Rusvikunin complex interacted with the purified human GPIIb/IIIa receptor to enhance the fluorescence intensity of the integrating proteins (Supplementary Fig. S9a,b). The Kd values of the interactions were 159.8 ± 8.9 nM and 184.9 ± 7.0 nM for the reconstituted and native complex, respectively (Fig. 6a,b), whereas the Kd value for fibronectin, a natural ligand for the platelet GPIIb/IIIa receptor, was 23.7 ± 0.9 nM (Supplementary Fig. S9c). Further, the custom peptides RusPep and cycRusPep also interacted with GPIIb/IIIa, though with higher Kd values (1.6 ± 0.09 µM and 0.8 ± 0.02 µM, respectively), compared to the Rusvikunin complex (Fig. 6c,d; Supplementary Fig. S9d,e). Thus, these findings corroborate the binding of the reconstituted/native Rusvikunin complex, Rusvikunin-II, cycRusPep, and RusPep to the GPIIb/IIIa receptor in α-chymotrypsin-treated platelets, leading to platelet aggregation.

### ELISA also reveals binding of the reconstituted and native Rusvikunin complex, RusPep, and cycRusPep to the GPIIb/IIIa receptor

**Figure 7:**
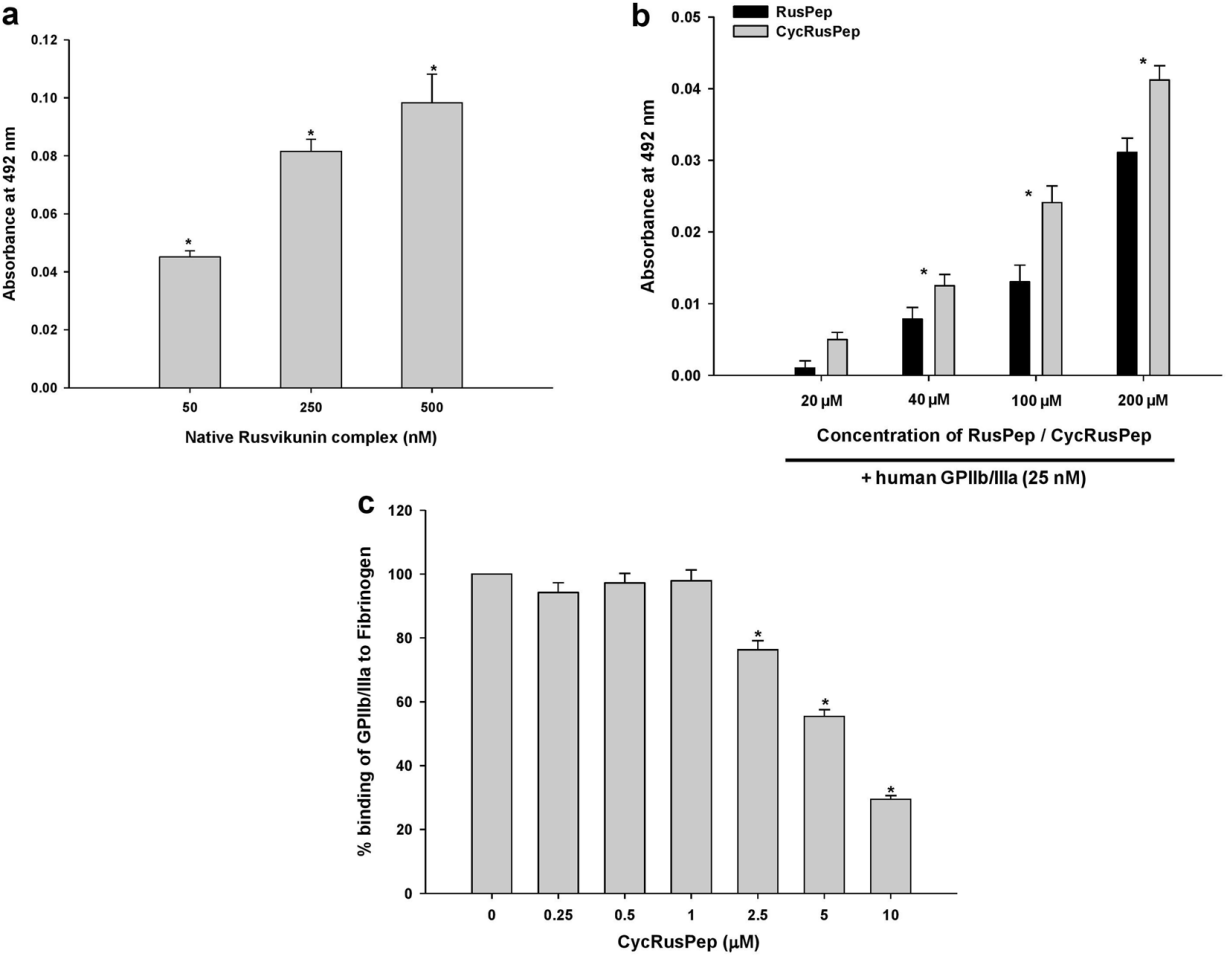
(**a**) Binding of native Rusvikunin complex with human GPIIb/IIIa receptors by ELISA. Experimental details are described in the text. The ELISA wells were coated with 50 to 500 nM of native Rusvikunin complex. (**b**) Binding of GPIIb/IIIa (500 ng) to ELISA wells coated with 20 to 200 µM of RusPep and cycRusPep. Values are mean ± SD of six determinations. Significance of difference with respect to control, *p < 0.05. (**c**) Inhibition of binding of human GPIIb/IIIa receptor to fibrinogen-coated ELISA plates by different concentration of cycRusPep (0.25–10 µM). Values are mean ± SD of six determinations. Significance of difference with respect to control, *p < 0.05.

ELISA studies suggested that the reconstituted/native Rusvikunin complex dose-dependently binds to the human GPIIb/IIIa receptor and vice-versa (Fig. 7a; Supplementary Fig. S10a,b). Nevertheless, compared to the Rusvikunin complex, fibronectin, and RusPep and cycRusPep demonstrated significantly higher (Supplementary Fig. S10c), and lower binding (Fig. 7b), respectively, to the GPIIb/IIIa receptor. These results indicate that the stretch of amino acids ^10^PESGRCRGHLRRIYYNLES^28^ of the Rusvikunin molecule, with its significant sequence similarity with fibronectin, is probably involved in the binding to the platelet GPIIb/IIIa receptor. Compared to the Rusvikunin complex, the lower binding of RusPep to GPIIb/IIIa may be due to their different 3-D conformations, which is in good agreement with *in silico* studies. Further, the ELISA competitive binding study demonstrated that cycRusPep dose-dependently inhibits GPIIb/IIIa binding to fibrinogen-coated wells (Fig. 7c), suggesting that the cyclic peptide that lacks an RGD motif can also bind to the GPIIb/IIIa receptor. This is in line with earlier findings of the inhibition of fibrinogen binding to the platelet GPIIb/IIIa receptor by the 29 kDa fragment (Ile^1359^ to Ser^1436^) of fibronectin and a 12-residue synthetic peptide ^1371^REDRVPHSRNSI^1382^ representing part of the Ile^1359^ to Ser^1436^ region of fibronectin that lacks the RGD motif^23,24^.

### Platelet modulation property of RusPep and cycRusPep

**Figure 8:**
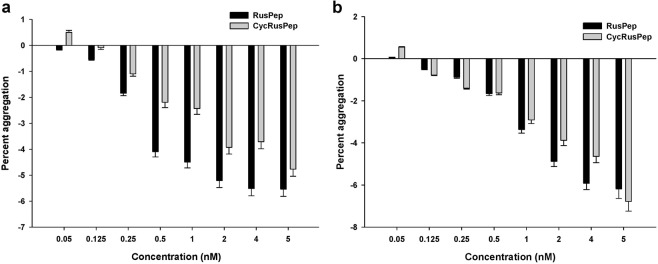
Concentration-dependent platelet deaggregation activity of RusPep and cycRusPep on (**a**). PRP and (**b**). Washed platelets isolated from goat blood. Values are mean ± SD of six determinations.

The customized peptides, RusPep, and cycRusPep at concentrations of 0.05–5.0 nM demonstrated concentration-dependent deaggregation of goat PRP and washed platelets (Fig. 8a,b), though the extent of platelet deaggregation was significantly lower (p < 0.05) than that of the reconstituted/native Rusvikunin complex or Rusvikunin-II. These results indicate the von Willebrand factor and/or fibrinogen-independent platelet deaggregation properties of RusPep and cycRusPep; however, both of the custom peptides failed to inhibit the collagen and ADP-induced platelet aggregation (data not shown).

Interestingly, although RusPep (5 nM) could bind to and interact with GPIIb/IIIa, it failed to aggregate α-chymotrypsin-treated washed platelets (Table 1). This suggests that the binding or interaction of a ligand with a certain receptor may not always translate into a response. On the contrary, cycRusPep (5 nM) could induce a marginal aggregation of α-chymotrypsin-treated washed platelets (Table 1). Therefore, the spatial arrangement of amino acids of the peptide ligands is also crucial for the binding and subsequent response induced by the ligand molecule, which is in good accordance with the findings of the molecular docking studies.

## Materials and Methods

Rusvikunin, Rusvikunin-II, and native Rusvikunin complexes were purified from the venom of Russell’s Viper (*D*. *russelii*) as described previously^15,16^. Briefly, crude *D*. *russelii* venom (450 mg protein) of Pakistan origin was subjected to gel filtration chromatography on a BioGel P-100 gel filtration column. The gel filtration fractions (from tube 131 to 135) were pooled, desalted, and then subjected to cation-exchange chromatography on a Tricorn MonoS 5/50 GL column connected to an AKTA Purifier Fast Protein Liquid Chromatography System (GE Healthcare, Sweden). The column was equilibrated with buffer A (20 mM 2-(N-morpholino)-ethanesulfonic acid, pH 6.0) for 10 min for eluting the unbound proteins, while the bound proteins were eluted with a linear gradient of buffer B (buffer A containing 1.0 M NaCl, pH 6.0) over 60 min. The Rusvikunin complex eluted as a sharp peak with 0.3 M NaCl^15^. The complex was further fractionated on a Jupiter C_18_ RP-HPLC column (250 mm × 4.6 mm) previously equilibrated with 0.1% (v/v) trifluoroacetic acid (TFA), on a Waters HPLC system. The column was first washed with 0.1% TFA for 10 min, thereafter the bound proteins were eluted with a linear gradient from 0 to 40% (v/v) acetonitrile (ACN) containing 0.1% (v/v) TFA over 75 min at a flow rate of 1 mL/min. Rusvikunin-II and Rusvikunin eluted at 34 and 44 min, respectively^15^. The Rusvikunin complex (~21 kDa) was reconstituted at 1:2 (Rusvikunin: Rusvikunin-II) ratio^17^. Sequencing-grade trypsin was procured from Promega, USA. Lyophilized polyvalent antivenom (raised against venom samples of *D*. *russelii*, *Naja naja*, *Echis carinatus*, and *Bungarus caeruleus*) was obtained from Bharat serum and Vaccines Ltd., India (Batch no. A05315029, expiry date: January 2019). The monoclonal antibodies against GPIIb/IIIa, P2Y_12_, and GPVI receptors were obtained from Santa Cruz Biotechnology Inc, USA. Human GPIIb/IIIa receptor was procured from Enzyme Research Laboratories, USA. Horseradish peroxidase (secondary antibody)-conjugated rabbit anti-mouse IgG and anti-horse IgG were procured from Sigma-Aldrich, USA. A linear peptide with 19 amino acid residues and a 7-amino acid cyclic (N to C) peptide representing regions of Rusvikunin-II, showing significant sequence homology with Ile^1359^ to Ser^1436^ residues of the fibronectin molecule were synthesized using Fmoc chemistry by GenPro Biotech Pvt. Ltd., Noida, India. The purity of the peptides (>98%) was established by reversed-phase HPLC. All other analytical grade chemicals were obtained from Sigma-Aldrich, USA.

### Platelet-modulating activity of Rusvikunin, Rusvikunin-II, and native and reconstituted Rusvikunin complex

Tezpur University Ethical Committee (TUEC) approved all the experimental protocols and the experiments were performed following the relevant guidelines and regulations of TUEC. Blood was collected from healthy volunteers (those who were not under medication) after obtaining informed consent from all the participants. Goat blood was collected from a slaughterhouse and used within 2 h of blood collection. Platelet-rich plasma (PRP) and the subsequent platelets were prepared from goat or human blood collected in 3.8% sodium citrate, according to the procedure of Bednar *et al*.^50^. The washed pellet was re-suspended in Tyrode’s buffer (137 mM NaCl, 5 mM HEPES, 12 mM NaHCO_3_, 2.7 mM KCl, 1 mM MgCl_2_, 0.42 mM, 0.1% glucose, Na_2_HPO_4_, and 0.25% bovine serum albumin) and the absorbance of the platelet suspension was adjusted using the same buffer to 0.15 at 540 nm^21^.

Different concentrations of reconstituted Rusvikunin complex (2.5–200 nM)/native Rusvikunin complex (2.5–200 nM)/Rusvikunin (7.5–600 nM)/Rusvikunin-II (7.5–600 nM) were mixed with 100 µL of pre-warmed PRP (at 37 °C) or washed platelet suspension in a 96-well microtiter plate for 5 s in a microplate reader (Multiskan GO, Thermo Scientific, Waltham, USA). Absorbance was then continuously recorded at 540 nm in a kinetic loop for 300 s at 15 s interval. Under identical conditions, the absorbance of PRP and PPP was also recorded and were subtracted from the experimental readings to determine the absorbance of platelets only in PRP. The percentage of platelet aggregation induced by different concentrations of agonist (Rusvikunin complex or Rusvikunin or Rusvikunin-II) was calculated according to the following formula, as described previously^14,49^:

1 $${\rm{Percent}}\,{\rm{aggregation}}=\frac{{\rm{Ao}}-{\rm{Af}}}{{\rm{Ao}}-{\rm{A}}}\times 100 \% $$

where Ao is the absorbance of PRP or platelet suspension at 540 nm prior to addition of agonist; Af is the absorbance of PRP or platelet suspension at 540 nm post addition of agonist; and A is PPP or Tyrode’s buffer absorbance at 540 nm^14,49^.

### Effect of native and reconstituted Rusvikunin complex/Rusvikunin-II on platelet aggregation induced by collagen and ADP

PRP (100 μL) or washed platelets (1 × 10^6^ cells/mL) was pre-incubated with different concentrations of agonists (2.5–600 nM) for 5 min followed by addition of type IV collagen (6 nM) or ADP (20 μM). The values were compared to those of collagen/ADP-induced aggregation that were considered as 100% activity^21^. Nevertheless, because Rusvikunin is a minor component of the complex, a limited amount of purified Rusvikunin was available and its effect on ADP and collagen-induced platelet aggregation could not be assayed.

In another set of experiments, 100 µL of washed platelets (1 × 10^6^ cells/mL) was pre-incubated with 10 μg/mL of monoclonal antibody (mAb) against GPVI (receptor for collagen)/P2Y_12_ (receptor for ADP) for 30 min before the addition of reconstituted Rusvikunin complex (2.5, 5.0, and 500 nM)/native Rusvikunin complex (2.5, 5.0, and 500 nM)/collagen type IV (6 nM)/ADP (20 μM)/PBS, pH 7.4 (control). The aggregation of platelets was measured as stated above.

### Effect on chymotrypsin-treated platelets

α-chymotrypsin was freshly prepared and the washed platelets (1 × 10^6^ cells/mL) were treated with 8 U/mL α-chymotrypsin/PBS, pH 7.4 (control) at room temperature (~23 °C) for 15 min followed by centrifugation for 15 min at 1500 × g^51^. The pellets were washed three times with Ca^2+^-free Tyrode’s buffer and finally re-suspended in the same buffer. Thereafter, human fibrinogen (175 nM)/reconstituted or native Rusvikunin complex (2.5 nM)/Rusvikunin II (7.5 nM) was added to the α-chymotrypsin-treated or control platelet suspension and the aggregation of platelets was measured as stated above. The platelet aggregation induced by BSA (1 µM), if any was also measured. The effect of monoclonal antibody against GPIIb/IIIa (a member of the integrin family of fibrinogen receptors) on Rusvikunin complex-induced platelet deaggregation or aggregation was assessed by pre-incubating α-chymotrypsin untreated platelets with 10 μg/mL mAb followed by addition of Rusvikunin complex (2.5 to 400 nM).

To confirm the binding of Rusvikunin complex or Rusvikunin-II to platelets treated with α-chymotrypsin, 10 μg/mL of monoclonal antibody against GPIIb/IIIa (member of the integrin family of fibrinogen receptors) was pre-incubated with washed platelets (1 × 10^6^ cells/mL) for 30 min at room temperature. Thereafter, the platelet aggregation was measured by adding human fibrinogen (175 nM)/reconstituted or native Rusvikunin complex (2.5 nM)/Rusvikunin-II (7.5 nM) to the α-chymotrypsin-treated platelets. The platelet aggregation induced by BSA (1 µM), if any was also measured.

### Platelet binding assay

Rusvikunin complex-specific antibodies were purified from equine polyvalent antivenom using a HiTrap NHS-Activated HP column (0.7 × 2.5 cm) connected to an AKTA Purifier 10 FPLC system (GE Healthcare, Sweden)^52^. Briefly, the immuno-affinity column was activated by sequentially passing 1 mM HCl (ice cold) followed by coupling buffer (200 mM NaHCO_3_ containing 500 mM NaCl, pH 8.3). Thereafter, the ligand (Rusvikunin complex) was dissolved in coupling buffer and coupled to the activated column for 4 h and the unbound proteins were then washed out. 100 mM Tris-HCl, pH 8.0 was used to block the non-reactive groups for overnight at 4 °C as per the manufacturer’s instructions. Thereafter, the column was subjected to repetitive alternate washes of high pH (100 mM Tris-HCl, pH 8.5) and low pH (100 mM acetate containing 500 mM NaCl, pH 4.0) buffers to deactivate any excess active groups. The column was equilibrated using 1X PBS, pH 7.4 and Indian polyvalent antivenom dissolved in the same buffer was applied to the column for binding for 2 h at 37 °C. Unbound antibodies were eluted with 1X PBS, pH 7.4 and then the Rusvikunin-specific bound antibodies were eluted with 100 mM glycine, pH 2.0 and neutralized immediately with 1 M Tris-HCl, pH 9.0. The neutralized antibody fraction was desalted using a PD_10_ column (GE Healthcare, Sweden), and the protein content was estimated^53^ and lyophilized for further use. The dilution of the specific antibody for optimum detection of the reconstituted and native Rusvikunin complex was determined by ELISA as stated below.

The binding of Rusvikunin-II, reconstituted and native Rusvikunin complexes to washed platelets was determined by ELISA. Briefly, platelets were coated onto the wells of a 96-well cell culture plate at 1 × 10^5^ platelets per well for overnight at 4 °C. Thereafter, the unbound platelets were removed by three washings for 5 min each with 1X PBS containing 0.05% tween-20 (wash buffer). After blocking the wells with 5% fat-free milk and washing thrice with the wash buffer, the wells were incubated with reconstituted or native Rusvikunin complex (250 nM) or Rusvikunin-II (750 nM) or 1X PBS, pH 7.4 (blank) at room temperature (~23 °C) for 30 min. After removing the excess Rusvikunin complex or Rusvikunin-II, by washing with the wash buffer, 100 µL of 1:500 dilutions of Rusvikunin complex-specific equine antibodies (1 mg/mL) was mixed to the wells and incubated at room temperature for 2 h. Unbound primary antibodies were removed by washing and then secondary antibody (rabbit anti-horse IgG conjugated to horseradish peroxidase) diluted to 1:2000 was added for detection of the bound primary antibodies. The reaction was incubated at room temperature for 2 h followed by addition of the substrate (1X TMB/H_2_O_2_) and incubation at room temperature in the dark. After 30 min 2 M H_2_SO_4_ was added to each well to stop the reaction and the color development was recorded at 492 nm against appropriate blanks in a microplate reader (Multiskan GO, Thermo Scientific, USA). The differential binding of native Rusvikunin complex (250–1000 nM) with goat or human washed platelets, if any, was also assessed by ELISA as described above.

In a separate set of experiments, well-bound platelets (1 × 10^6^ cells/mL) were incubated with 8 U/mL α-chymotrypsin/PBS, pH 7.4 (control) at room temperature (~23 °C) for 5–15 min^37,38^. After incubation, the wells were washed three-times with wash buffer. Subsequently, three wells were incubated with 10 µg/mL of monoclonal antibody against GPIIb/IIIa receptor, and the remaining wells with PBS, pH 7.4. After 30 min incubation at room temperature, the wells were washed with wash buffer and then reconstituted or native Rusvikunin complex (250 nM) or Rusvikunin-II (750 nM) was added to each well and further incubated at room temperature for 30 min. After washing off the unbound Rusvikunin complex or Rusvikunin-II, the bound proteins were detected by ELISA as described above.

### *In silico* analysis to determine the RGD-independent binding region in Rusvikunin-II

Fibronectin is reported to bind to the GPIIb/IIIa receptor independently of the RGD domain. Therefore, the amino acid stretch of fibronectin (Ile^1359^ to Ser^1436^) that shows RGD-independent binding was aligned with Rusvikunin-II (the major component of Rusvikunin complex) by a pair-wise sequence alignment using EMBOSS Needle (Global alignment) and EMBOSS Water (Local alignment) servers of the European Bioinformatics Institute (EMBL-EBI). The primary sequence of Rusvikunin-II that showed a considerable sequence homology with the Ile^1359^ to Ser^1436^ residues of the fibronectin molecule was used to synthesize linear (RusPep) and cyclic (N to C cyclised) (cycRusPep) custom peptides. The molecular mass and purity of the synthetic peptides were judged by MALDI-ToF-MS and RP-HPLC analyses. The effect of different doses of RusPep (0.05–5 µM) and cycRusPep (0.05–5 µM) on goat PRP/washed platelets/α-chymotrypsin-treated washed platelets was determined as described above.

The complete sequences of human GPIIb (P08514) and GPIIIa (P05106) subunits were retrieved from the UniProt database. Rusvikunin-II showed significant identity (42% coverage) with KSPI 2 (accession no. P00900) from *D*. *siamensis*^16^, and therefore, the amino acid sequence of this KSPI was considered for modeling. For structure prediction, all of these primary sequences were deposited to the I-TASSER server^54^. The best model structures of human GPIIb, GPIIIa, and Rusvikunin-II were then selected based on Tm values and C-scores. The best models of human GPIIb and GPIIIa were docked using the PatchDock server^55^ and further refined with the Firedock server^56^. The refined structure of human GPIIb/IIIa was then docked with the best model of Rusvikunin-II (as predicted by the I-TASSER server) using the PatchDock server and further refined with the Firedock server. The resulting structure was then submitted to the PDBSum online server to determine the residue-to-residue interactions between human GPIIb/IIIa and Rusvikunin-II. The structures of the two custom peptides (RusPep and CycRusPep) were predicted using the PEP-FOLD3 and PEPstrMOD peptide structure prediction online web servers, respectively^57,58^. The best structures of these custom peptides were then independently docked with the refined structure of human GPIIb/IIIa using the PatchDock server and further refined with the Firedock server. The residue-to-residue interactions between human GPIIb/IIIa and the custom peptides were then determined using the PDBSum online server.

### Assessment of binding of reconstituted and native Rusvikunin complex, RusPep, and cycRusPep with human GPIIb/IIIa by ELISA

The binding of native/reconstituted Rusvikunin complex, and RusPep/cycRusPep to human GPIIb/IIIa was evaluated by ELISA^8^. In the first set of experiments, 100 µL of 50–500 nM reconstituted or native Rusvikunin complex (100–1000 ng) and 20–200 µM RusPep/cycRusPep (1500–50000 ng) was coated onto the wells of an ELISA plate. After removing the unbound proteins/peptides from the wells and blocking with 5% fat-free milk, 100 µL of 25 nM human GPIIb/IIIa (500 ng)/1X PBS, pH 7.4 (control) was mixed and allowed to stand at room temperature for 4 h. Subsequently, 100 µL of anti-GPIIb/IIIa antibody (primary antibody raised in mouse; 1:100 dilutions) was added and incubated at room temperature for 2 h. Detection was done by incubating the wells with rabbit anti-mouse IgG-HRP conjugate secondary antibody (1:2000 dilutions) for 2 h and the substrate (1X TMB/H_2_O_2_) was then added for the color development that was measured at 492 nm. For the above experiment, positive controls were assessed in wells that were coated with fibronectin (100–1000 ng).

In another set of experiments, wells were coated with 100 µL of 25 nM human GPIIb/IIIa (500 ng). They were then incubated with 100 µL of 500–1000 nM reconstituted or native Rusvikunin complex (1000 and 2000 ng) for 4 h at room temperature. The affinity purified Rusvikunin complex-specific antibodies (1:500 dilutions of 1 mg/mL stock) served as the primary antibody and the secondary antibody was anti-horse IgG-HRP (1:2000 dilutions). Detection was carried out as described above.

### Competitive binding assay of cycRusPep and human GPIIb/IIIa with human fibrinogen by ELISA

One hundred microlitres of 30 nM fibrinogen (1000 ng) were coated to the wells of an ELISA plate at 4 °C for overnight, and then the excess unbound fibrinogen was washed off with wash buffer. Thereafter, 100 µL of 25 nM human GPIIb/IIIa (500 ng) was incubated with 100 µL of 0.25–10 µM cycRusPep (20–1000 ng) for 4 h at room temperature and the mixture was then transferred to the fibrinogen-coated wells and further incubated at room temperature for 2 h. The primary anti-GPIIb/IIIa antibody (1:100 dilution) was then added to the wells and detected with the secondary antibody (rabbit anti-mouse IgG-HRP conjugate) at 1:2000 dilution. Thereafter, 1X TMB/H_2_O_2_ was added for the color development, and the final reaction product was determined at 492 nm.

### Determination of interaction between Rusvikunin complex/custom peptides and human GPIIb/IIIa by spectrofluorometry analysis

The protein-protein and protein-peptide interactions between the human GPIIb/IIIa receptor and RusPep/cycRusPep/reconstituted or native Rusvikunin complex/fibronectin (positive control) was analyzed by the spectrofluorometry titration method^17,20^ using a fluorescence spectrometer (LS 55, Perkin Elmer, Palo Alto, CA). Increasing concentrations of RusPep (0.5–10 µM)/cycRusPep (0.5–10 µM)/reconstituted or native Rusvikunin complex (35–700 nM)/fibronectin (7.5–75 nM) were added to fixed concentrations of human GPIIb/IIIa receptor (6 nM) in a quartz cuvette at 23 °C and the mixture was excited at 280 nm. The emission spectrum was measured between 300–400 nm wavelengths. The slit length was maintained at 10 nm. The one site-specific binding model (equation 2) was used to determine the Kd values of the interactions in the GraphPad Prism 5.0 software.

2 $$\Delta F=\frac{\Delta Fmax\times C}{Kd+C}$$

where ΔF is the change in fluorescence intensity of GPIIb/IIIa in the presence of RusPep/cycRusPep/reconstituted or native Rusvikunin complex/fibronectin; ΔFmax is the maximum change in fluorescence intensity of GPIIb/IIIa when saturated with RusPep/cycRusPep/reconstituted or native Rusvikunin complex/fibronectin; and C is the concentration of RusPep/cycRusPep/reconstituted or native Rusvikunin complex/fibronectin.

### Statistical analysis

All experiments contained a minimum of three and up to six replicates. Results are depicted as means and the standard deviation of the means are represented by the error bars. The significance of difference between means of test samples and control was determined using Student’s *t*-test in Sigma Plot 11.0 for Windows (version 10.0). A value of p ≤ 0.05 was considered significant.

## Supplementary information

Supplementary Tables and Figures

## Data Availability

Authors confirm that all relevant data are included in the paper and/or its supplementary information file.
