# Supplementary material for: RGD-independent binding of Russell’s Viper venom Kunitz-type protease inhibitors to platelet GPIIb/IIIa receptor
Source: Sci Rep. 2019 Jun 5;9:8316. doi: 10.1038/s41598-019-44767-2 (PMC6549151; doi:10.1038/s41598-019-44767-2)
Supplement: Supplementary file 1 — Supplementary Tables and Figures [file 41598_2019_44767_MOESM1_ESM.docx]

**RGD-independent binding of Russell’s Viper venom Kunitz-type protease inhibitors to platelet GPIIb/IIIa receptor**

**Bhargab Kalita, Sumita Dutta, Ashis K. Mukherjee***

Microbial Biotechnology and Protein Research Laboratory, Department of Molecular Biology and Biotechnology, Tezpur University, Tezpur 784028, Assam, India

*Corresponding author: Dr. A. K. Mukherjee, Department of Molecular Biology and Biotechnology, Tezpur University, Tezpur 784028, Assam, India. Ph: +913712275405; E-mail: [akm@tezu.ernet.in](mailto:akm@tezu.ernet.in)

**Supplementary Figures**

**Figure S1a.** Concentration-dependent platelet modulating activity of reconstituted Rusvikunin complex on PRP from goat blood. Values are mean ± SD of triplicate determinations.

**Figure S1b.** Concentration-dependent platelet modulating activity of Rusvikunin and Rusvikunin-II on PRP from goat blood. Values are mean ± SD of triplicate determinations.

**Figure S1c.** Concentration-dependent platelet modulating activity of reconstituted Rusvikunin complex on PRP from human blood. Values are mean ± SD of triplicate determinations.

**Figure S1d.** Concentration-dependent platelet modulating activity of Rusvikunin and Rusvikunin-II on PRP from human blood. Values are mean ± SD of triplicate determinations.

**Figure S2.** Binding of native Rusvikunin complex (250 to 1000 nM) with human and goat washed platelets by ELISA. Values are mean ± SD of six determinations. Significance of difference with respect to binding to human platelets, *p < 0.05.

**Figure S3.** Concentration-dependent platelet modulating activity of reconstituted Rusvikunin complex on washed platelets from goat blood. Values are mean ± SD of triplicate determinations.

**Figure S4.** Inhibition of Rusvikunin complex-induced platelet aggregation and deaggregation by anti GPIIb/IIIa mAb. The Rusvikunin complexes induced platelet deaggregation at 2.5 and 5.0 nM, while they caused aggregation of platelets at 200 and 400 nM. Values are mean ± SD of triplicate determinations.

**Figure S5.** Determination of binding of reconstituted Rusvikunin complex (250 nM) or Rusvikunin-II (750 nM) on α-chymotrypsin-treated or untreated washed platelets (1 × 10^6^ cells/mL) by ELISA. Experimental details are described in the text. Values are mean ± SD of six determinations. Significance of difference with respect to Native platelets, *p < 0.05.

**Figure S6a.** Effect of different concentrations of Rusvikunin-II (7.5-600 nM) on ADP (20 μM)-induced aggregation of goat and human PRP. Values are mean ± SD of six determinations.

**Figure S6b.** Effect of different concentrations of reconstituted Rusvikunin complex (2.5-200 nM) on ADP (20 μM)-induced aggregation of goat and human PRP. Values are mean ± SD of six determinations.

**Figure S6c.** Effect of ADP (20 μM) or reconstituted Rusvikunin complex (500 nM) on human washed platelets (1 × 10^6^ cells/mL) pre-incubated with monoclonal antibody (10 μg/mL) against ADP P2Y_12_ receptor or buffer (1X PBS, pH 7.4) for 30 min at ~23°C. The monoclonal antibody did not induce platelet aggregation. Values are mean ± SD of six determinations. Significance of difference with respect to platelet aggregation in presence of mAb against P_2_Y_12_ receptor, *p < 0.05.

**Figure S7a.** Dose dependent effect of reconstituted Rusvikunin complex (2.5-100 nM) on collagen (6 nM)-induced aggregation of goat and human PRP. The aggregation induced by collagen was considered as 100% aggregation and the other values were compared to that. Values are mean ± SD of six determinations. Significance of difference with respect to platelet aggregation induced by collagen only; *p<0.05.

**Figure S7b.** Effect of collagen (6 nM) or reconstituted Rusvikunin complex (500 nM) on human washed platelets (1 × 10^6^ cells/mL) pre-incubated with monoclonal antibody (10 μg/mL) against collagen GPVI receptor or buffer (1X PBS, pH 7.4) for 30 min at ~23°C. The monoclonal antibody did not induce platelet aggregation. Values are mean ± SD of six determinations. Significance of difference with respect to platelet aggregation in presence of mAb against GPVI receptor, *p < 0.05.

**Figure S8.** Alignment of Rusvikunin sequence with fibronectin region Ile^1359^ to Ser^1436^ using **a.** Emboss Needle (Global alignment; Needleman-Wunsch algorithm) and **b.** Emboss Water (Local alignment, Smith-Waterman algorithm).

**Figure S9a.** Spectrofluorometry analysis to study the interaction of human GPIIb/IIIa (6 nM) with native Rusvikunin complex (35-700 nM).

**Figure S9b.** Spectrofluorometry analysis to study the interaction of human GPIIb/IIIa (6 nM) with reconstituted Rusvikunin complex (35-700 nM).

**Figure S9c.** Spectrofluorometry analysis to study the interaction of human GPIIb/IIIa (6 nM) with fibronectin (7.5-75.0 nM). **Inset:** One site-specific binding curves showing the change in the maximum fluorescence intensity (λmax) of human GPIIb/IIIa (6 nM) in presence of different concentrations of fibronectin (7.5-75.0 nM).

**Figure S9d.** Spectrofluorometry analysis to study the interaction of human GPIIb/IIIa (6 nM) with RusPep (0.6-12 µM).

**Figure S9e.** Spectrofluorometry analysis to study the interaction of human GPIIb/IIIa (6 nM) with cycRusPep (0.5-10 µM).

**Figure S10a.** Binding of reconstituted Rusvikunin complex with human GPIIb/IIIa receptors by ELISA. Experimental details are described in the text. The ELISA wells were coated with 50 to 500 nM of reconstituted Rusvikunin complex. Values are mean ± SD of six determinations. Significance of difference with respect to control, *p < 0.05.

**Figure S10b.** Binding of 500 and 1000 nM of reconstituted and native Rusvikunin complex to ELISA wells coated with 25 nM (500 ng) of GPIIb/IIIa. Values are mean ± SD of six determinations. Significance of difference with respect to control, *p < 0.05.

**Figure S10c.** Binding of GPIIb/IIIa (500 ng) to ELISA wells coated with 100-1000 ng of fibronectin. Values are mean ± SD of six determinations. Significance of difference with respect to control, *p < 0.05.

**Supplementary Table S1:** Percent aggregation of washed platelets (1 × 10^6^ cells) by different agonists in presence of Native Rusvikunin complex. Values are mean ± SD of three determinations.

| **Agonist** | **mAb (10 µg/mL)** | **Native Rusvikunin complex** | **% platelet aggregation** | **% inhibition by mAb** |
| --- | --- | --- | --- | --- |
| Collagen (6 nM) | - | 0 nM | 26.2 ± 0.84 | - |
|  | - | 2.5 nM | 6.6 ± 0.21 | - |
|  | - | 5.0 nM | 7.9 ± 0.12 | - |
|  | anti GPVI | 2.5 nM | 1.3 ± 0.04 | 80.3 ± 2.1 |
|  | anti GPVI | 5.0 nM | 0.8 ± 0.01 | 90.1 ± 2.4 |
| ADP (20 µM) | - | 0 nM | 15.7 ± 0.64 | - |
|  | - | 2.5 nM | (-)10.7 ± 0.21 | - |
|  | - | 5.0 nM | (-)20.5 ± 0.06 | - |
|  | anti P2Y_12_ | 2.5 nM | (-)1.3 ± 0.04 | 87.5 ± 2.2 |
|  | anti P2Y_12_ | 5.0 nM | (-)2.6 ± 0.02 | 87.3 ± 2.6 |
